# Molecular analysis of mitochrondrial *cytb* of *Pediculus humanus capitis* in Thailand revealed potential historical connection with South Asia

**DOI:** 10.1371/journal.pone.0257024

**Published:** 2021-09-07

**Authors:** Kobpat Phadungsaksawasdi, Sakone Sunantaraporn, Nirin Seatamanoch, Switt Kongdachalert, Atchara Phumee, Kanyarat Kraivichian, Vorthon Sawaswong, Sunchai Payungporn, Narisa Brownell, Padet Siriyasatien

**Affiliations:** 1 Department of Parasitology, Faculty of Medicine, Chulalongkorn University, Bangkok, Thailand; 2 Vector Biology and Vector Borne Disease Research Unit, Department of Parasitology, Faculty of Medicine, Chulalongkorn University, Bangkok, Thailand; 3 Department of Medical Technology, School of Allied Health Sciences, Walailak University, Nakhon Si Thammarat, Thailand; 4 Program in Bioinformatics and Computational Biology, Graduate School, Chulalongkorn University, Bangkok, Thailand; 5 Department of Biochemistry, Faculty of Medicine, Chulalongkorn University, Bangkok, Thailand; Banaras Hindu University Faculty of Science, INDIA

## Abstract

**Background:**

*Pediculus humanus capitis* or head louse is an obligate ectoparasite and its infestation remains a major public health issue worldwide. Molecular analysis divides head lice into six clades and intra-clade genetic differences have been identified. Several hypotheses have been formulated to elucidate the discrepancies of the variety of head lice among different regions of the world. It is currently concluded that head lice distribution might be associated with human migration history. This study aims to investigate genetic data of human head lice in Thailand. We believe that the analysis could help establish the correlation between local and global head lice populations.

**Method:**

We investigated mitochondrial cytochrome b (*cytb*) gene of the collected 214 head lice to evaluate genetic diversity from 15 provinces among 6 regions of Thailand. The head lice genes were added to the global pool for the phylogenetic tree, Bayesian tree, Skyline plot, and median joining network construction. The biodiversity, neutrality tests, and population genetic differentiation among the 6 Thailand geographic regions were analyzed by DNAsp version 6.

**Results:**

The phylogenetic tree analysis of 214 collected head lice are of clade A and clade C accounting for roughly 65% and 35% respectively. The Bayesian tree revealed a correlation of clade diversification and ancient human dispersal timeline. In Thailand, clade A is widespread in the country. Clade C is confined to only the Central, Southern, and Northeastern regions. We identified 50 novel haplotypes. Statistical analysis showed congruent results between genetic differentiation and population migration especially with South Asia.

**Conclusions:**

Pediculosis remains problematic among children in the rural areas in Thailand. *Cytb* gene analysis of human head lice illustrated clade distribution and intra-clade diversity of different areas. Our study reported novel haplotypes of head lice in Thailand. Moreover, the statistic calculation provided a better understanding of their relationship with human, as an obligate human parasite and might help provide a better insight into the history of human population migration. Determination of the correlation between phylogenetic data and pediculicide resistance gene as well as residing bacteria are of interest for future studies.

## Introduction

The head louse is an obligate human-ectoparasite causing pediculosis [1]. Pediculosis affects people of all demographics globally [2]. *Pediculus humanus capitis* is categorized as Phthiraptera (lice), suborder Anoplura, which is also known as sucking lice. Anoplura is divided into two genera; Pthirus and Pediculus. The latter genus contains medically important species, *Pediculus humanus*. *Pediculus humanus* are grouped into two kinds; head lice (*Pediculus humanus capitis*) and body lice (*Pediculus humanus humanus*). Human head lice feed on human blood. Its blood-sucking behavior can result in severe scalp pruritis and on-top skin bacterial infections [3]. Treatment options are mainly focused on topical agents and mechanical devices, while per oral ivermectin is the treatment of choice in refractory cases [2]. To date, there is a rise in the resistance against widely used pediculicides [4–6].

Multiple pathogenic bacteria were unintentionally detected in lice. There is a solid evidence that body louse is a vector of fatal diseases, such as epidemic typhus, trench fever, and relapsing fever [7]. However, more information is required to support the role of head lice as a disease spreader despite reports of both pathogenic and non-pathogenic microorganisms detection, such as *Bartonella quintana*, *Borrelia recurrentis*, and *Acinetobactor* spp. [8].

Genetic analysis greatly sheds light on the molecular study of lice [9]. Lice ancestor tracking is currently possible due to the advent of molecular investigations. Not only does the knowledge acquired help explain the development of head lice and body lice itself, but it also provides additional independent clues of human evolution [10, 11]. To exemplify, Tree map 2.0 (Reconciliation Analysis) confirmed that pediculus diverged into *Pediculus schaeffi* and *Pediculus humanus* roughly 5.6 million years ago (MYA). Of note, *Pediculus schaeffi* occurs exclusively on chimps while *Pediculus humanus* occurs only in human being. This information correlates with the previous report regarding genetic split of chimpanzee and human approximately 6 MYA, thus verifying their coevolutionary association. Evidence of head lice infestations thousands of years ago have been obtained by a study that collected head lice from mummies of the North of Chile [12]. The mummies’ ages were estimated to be from 2000 BC (Before Christ) and 500 AD (Anno Domini). Another study by Raoult et al. collected head lice from mummies of the calibrated age 1025 years old excavated from Peruvian coastal area [13]. The molecular study was conducted to study their clades.

Mitochondrial DNAs of bilateral animals are generally in a circular architecture. However, in head and body lice, the genetic material is in the form of small linear fragments extending 3–4 kb [14]. The riddles of the evolution and the consequences of this phenomenon remain to be solved. Molecular studies of two mitochondrial genes; cytochrome oxidase I (*COXI*) and cytochrome b (*cytb*) found that *Pediculus humanus* can be divided into deep distinct lineages. Lice clades were initially divided into three groups; A, B, and C, but the study was geographically limited to Europe and the New World [15]. Later studies expanded its regional specimen collection and revealed clade D, E, and F [16, 17]. Interestingly, it has been observed that lice of different clades occurred on human of different areas illustrating the timeline of emerging head lice as well as patterns of human migration [9]. Clade A is globally distributed, while others are restricted to certain regions [18]. Clade B originated in North America [15]. Clade C is merely found in Africa and Asia [16, 19]. Clade D has been reported only in sub-Saharan African countries and Malaysia and is a sister clade of clade A [20–22]. Clade E, the sister clade of clade C, has been found in Ethiopia [11, 18]. Clade F, the sister group of clade B, is the most recent clade to be reported. It was first discovered in French Guyana and was also described in Argentina and Mexico [17].

So far, molecular analysis digs deeper into intra-clade diversities [15, 17, 18, 23–25]. Molecular examination of mitochondrial genes further describes the heterogeneity of head lice in haplotypes. The haplotype with the highest global population is A5 for clade A and B36 for clade B [18, 23]. Recent papers added up to the pool of global lice haplotypes [26, 27]. Assumptions have been made that the area with a higher diversity of haplotypes has a higher tendency to be the origin of the clade, therefore the findings assist in clarifying head lice and human migration and evolution [15].

This study is the first to survey the genetic data of human head lice collected from different geographical regions of Thailand using *cytb* gene. The objective is to provide deeper molecular epidemiological information as well as evolutional knowledge of Thailand head lice.

## Materials and methods

### 1. Ethical approval

The study was approved and reviewed by the Institutional Review Board of the Faculty of Medicine, Chulalongkorn University in Bangkok, Thailand (COA no.1352/2020). The study was explained to each participant and written consent was filled out by each parent or guardian.

### 2. Head louse collection

Head lice DNA samples in the study were derived from the previous study by Sunantaraporn et al. [16]. A total of 214 head lice were obtained between Jan. 2014 and Dec. 2014 from students of 26 primary schools in 15 of 77 provinces throughout six geographical regions in Thailand including Northern (Chiang Rai, Nan, and Phrae), Northeastern (Loei, Mukdahun, and Nakhon Ratchasima), Central (Bangkok, Lopburi, Nakhon Pathom, and Saraburi), Western (Kanchanaburi), Eastern (Rayong), and Southern (Nakhon Si Thammarat, Phatthalung, and Songkhla) regions. The inclusion criteria were as follows; female school children, age from 7 to 12 years, at least one head louse detected by a fine-tooth comb. The exclusion criteria were concurring scalp diseases and disorders, or denial to give informed consent. Each participant from randomly selected primary school was recruited by research staff including entomology laboratory analysts and medical entomologists using cluster sampling method. The sampling method utilized was considered extrapolative to the population as cluster sampling is a probability sampling and thus claimed suitable for the study of head lice demographics in the country. A total of 209 individual adult head lice and 5 samples of head lice eggs were collected using standard fine-tooth comb. Adult head lice were identified morphologically under light microscopy. These clinical specimens were preserved in 70% ethanol at 4°C and were transferred to Vector Biology and Vector Borne Disease Research Unit, Department of Parasitology, Faculty of Medicine, Chulalongkorn University for further molecular analysis.

### 3. DNA extraction

Head lice samples were rinsed 3 times with 1X phosphate buffer saline (PBS) and stored dry at room temperature. Head lice DNA was lysed by 200 μl lysis buffer G and 20 μl of proteinase S, and then grounded with a sterile plastic pestle. Genomic DNA was extracted by DNA extraction kit, Invisorb® spin tissue mini kit (STRATEC molecular GmbH, Berlin, Germany), according to the manufacturer’s protocol. Fifty microlitres of elution buffer was used to elute the extracted DNA. The purified DNA concentration was measured by Nano drop 2000c (Thermo-scientific, USA), and then was kept at -20°C.

### 4. *cytb* gene amplification by PCR

Standard PCR was conducted to amplify the 348 bp mitochondrial *cytb* gene as mentioned in a previous study by Li et al. [28]. The sequence of the primers designed were CytbF (5’- GAGCGACTGTAATTACTAATC-3’) and CytbR (5’-CAACAAAATTATCCGGGTCC-3’) as forward and reverse primers respectively. PCR mixture of the amplification was performed in a total volume of 25 μl, consisting of 3 μl of extracted DNA, 10X PCR buffer, 25 mM of MgCl_2_, 2.5 mM of dNTPs, 10 μM of each forward and reverse primers, and 1 U of *Taq* DNA polymerase (Thermo Scientific, Waltham, MA, USA). The PCR condition profile was as follows; pre-denaturation at 95°C for 3 minutes; followed by 35 cycles including 30 sec denaturation at 95°C, 30 sec annealing at 53°C, 1 min extension at 72°C following by 7 min final extension at 72°C. PCR was carried out in a PCR Mastercycler Pro S (Eppendorf, Germany). Sterile distilled water and plasmid DNA containing *cytb* gene of *P*. *h*. *capitis* strain was used as negative and positive control respectively. These PCR amplicons were separated via electrophoresis on 1.5% agarose gel which were stained with ethidium bromide and illustrated under Quantity One Quantification Analysis Software version 4.5.2 (Gel DocEQ System; BioRad, Hercules, CA).

### 5. Nucleotide cloning and sequencing

The PCR products were cloned into pGEM-T Easy Vector (Promega, Madison, WI) using DNA ligation kit (Promega, Madison, WI). Five microlitres of ligated DNA were transformed into *Escherichia coli* DH5α competent cells. The chimeric DNA was screened using the blue-white colonies selection system. Transformed cells were cultured in Luria-Bertani (LB) broth with ampicillin (100 mg/ml). Chimeric DNA was isolated using the Invisorb® Spin Plasmid Mini kit (STRATEC molecular GmbH, Berlin, Germany) following the manufacturer’s instructions. The purified plasmids were sequenced by the commercial service (Macrogen Inc., South Korea).

### 6. Statistical analysis

The obtained *cytb* sequences were aligned and edited using BioEdit Sequence Alignment Editor Version 7.2.5. All *cytb* sequences were acquired by nucleotide sequence identity searches using Basic Local Alignment Search Tool (BLAST) (https://blast.ncbi.nlm.nih.gov/Blast.cgi). All of the sequences were compared with the sequences available in the GenBank database. The sequences of the head lice collected in our study were pooled together with global dataset provided by Amanzougaghene to perform phylogenetic tree and haplotype analysis. The current global library (excluding our data) contains 1,883 samples from 54 countries of 6 continents around the world. (Africa: 1,029, Asia: 264, Europe: 239, Oceania: 91, South America: 176, North America: 84). The phylogenetic tree was constructed by the maximum likelihood (ML) method using HKY+G substitution model implemented in MEGA X software. The reliability of the phylogenetic tree was estimated by performing 1000 bootstrap replicates. *Pediculus schaeffi* accession no. AY696067 was an outgroup. *Pediculus humanus capitis cytb* haplotype network was constructed using PopART software [29]. Median-joining network is generally used to infer intraspecific phylogenies [30]. The biodiversity and neutrality tests were computed by DNAsp version 6 to calculate for number of haplotypes (H), number of polymorphic sites (S), average number of nucleotide differences (k), haplotype diversity (Hd), nucleotide diversity (π), Tajima’s D, and Fu and Li’s D. The pairwise analysis was done between each of the six regions in Thailand: Central, North, Northeast, South, West, and East resulting in a total of 15 comparisons [31]. Population genetic differentiation using pairwise Kst was also calculated by DNAsp version 6.

#### Bayesian phylogenetic analysis and skyline plot analysis

A total of 165 *cytb* unique haplotypes were subsequently used for Bayesian phylogenetic inference and divergence time analysis using BEAST2 software (version 2.6.4) [32]. A sequence of *P*. *schaeffi* (AY696067) was included to calibrate the divergent time point splitting between *P*. *schaeffi* and *P*. *humanus* at 6 million years ago (6 ± 0.5) [11].

The data were sub-analyzed into 2 partitions. The first partition was analyzed with the parameter estimation across all codons while the second partition allowed the varied parameter estimation across each codon position. The HKY + G substitution model was implemented in analysis performed under a lognormal relaxed clock model.

The calibration priors were estimated as normal distributions. Tree prior was calculated under Yule speciation model. The Markov chain Monte Carlo (MCMC) analysis was run for 30 million generations sampling every 2000 generations. The maximum clade credibility tree was constructed using Tree Annotator (implemented in BEAST2 package) by discarding initial 10% data as burn-in. The tree was configured and edited in FigTree software (version 1.4.4). The coalescent Bayesian skyline analysis were conducted using the same model of phylogenetic analysis but include only one partition estimating the parameter across all codons [33]. The skyline plot was constructed by Tracer (version 1.7.1) and plotted by GraphPad Prism 7 software.

## Results

### Subject demographics

All 214 participants were female elementary school students who were of the ages of 7 to 12. The mean age was 9.22 ± 2.18 years. The data regarding their age and geographical location were displayed in Table 1.

**Table 1:** Demographic characteristics of primary school children with head lice infestation in Thailand.

Characteristics	Frequency (n)	Percentage (%)
**Age**		
7	44	20.56
8	32	14.95
9	37	17.29
10	45	21.03
11	45	21.03
12	11	5.14
Total	214	100
**Sex**		
Female	214	100
Total	214	100
**Geographical Region**		
Central	35	16.36
Northern	15	7.00
Northeastern	45	21.03
Southern	66	30.84
Western	42	19.63
Eastern	11	5.14
Total	214	100

### Sequencing, phylogenetic tree analysis, Bayesian tree analysis, skyline plot analysis, and median joining network

Our study collected 214 specimens from 15 provinces of all 6 geographic regions in Thailand. For the phylogenetic tree analysis, we used all unique sequences detected in Thailand together with all unique entries available in GenBank. The country/continent in which the specific haplotype was reported was attached at the end of the sequence (for example, *Pediculus humanus capitis* Hap. A16 Asia, Oceania). In regards with median joining network creation, we used all global entries combined with all of the collected entries from our current study. The number of all sequences combined was 2097 sequences. Genetic population analysis was focused on our country. The sequences utilized were of our study (214 samples).

All sequences of *P*. *h*. *capitis* collected from our study, when compared with the data in Genbank, revealed close linkage to *Pediculus humanus capitis*. The percent identity ranged from 98.63% to 100%. Our head lice genetic data was identical with 5 haplotypes of *Pediculus humanus capitis*: A5, A16, A17, A67, and C41 (Accession No. MN515380, KM579552, MN515374, MH230927, KJ840551). The obtained *cytb* sequences in the study were submitted in the Genbank database under the accession numbers; MW091050-MW091263.

To date, *Pediculus humanus capitis* molecular determination using *cytb* successfully defined deeply divergent six clades; A, B, C, D, E, and F. *Cytb* gene consists of 272 base pairs.

**Fig 1:**
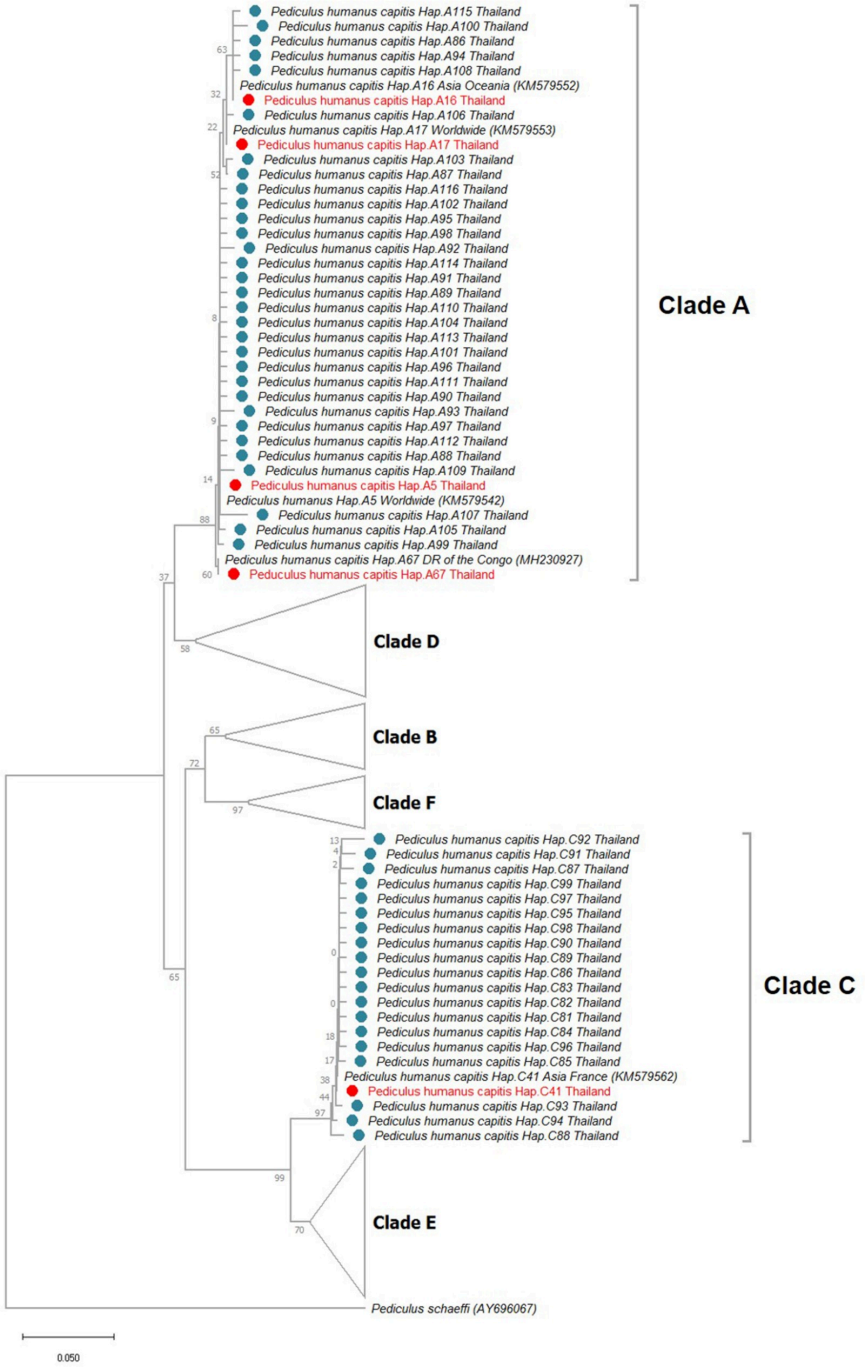
Phylogenetic tree analysis. The ML tree illustrates 6 clades. A total of 2097 sequences consisting of the global data and our newly obtained were used for the analysis. The sequences were from North America, South America, Europe, Africa, Asia, and Oceania. Clade A and C were the focus as they were found in Thailand. Other clades have never been reported in the country yet.

**Fig 2:**
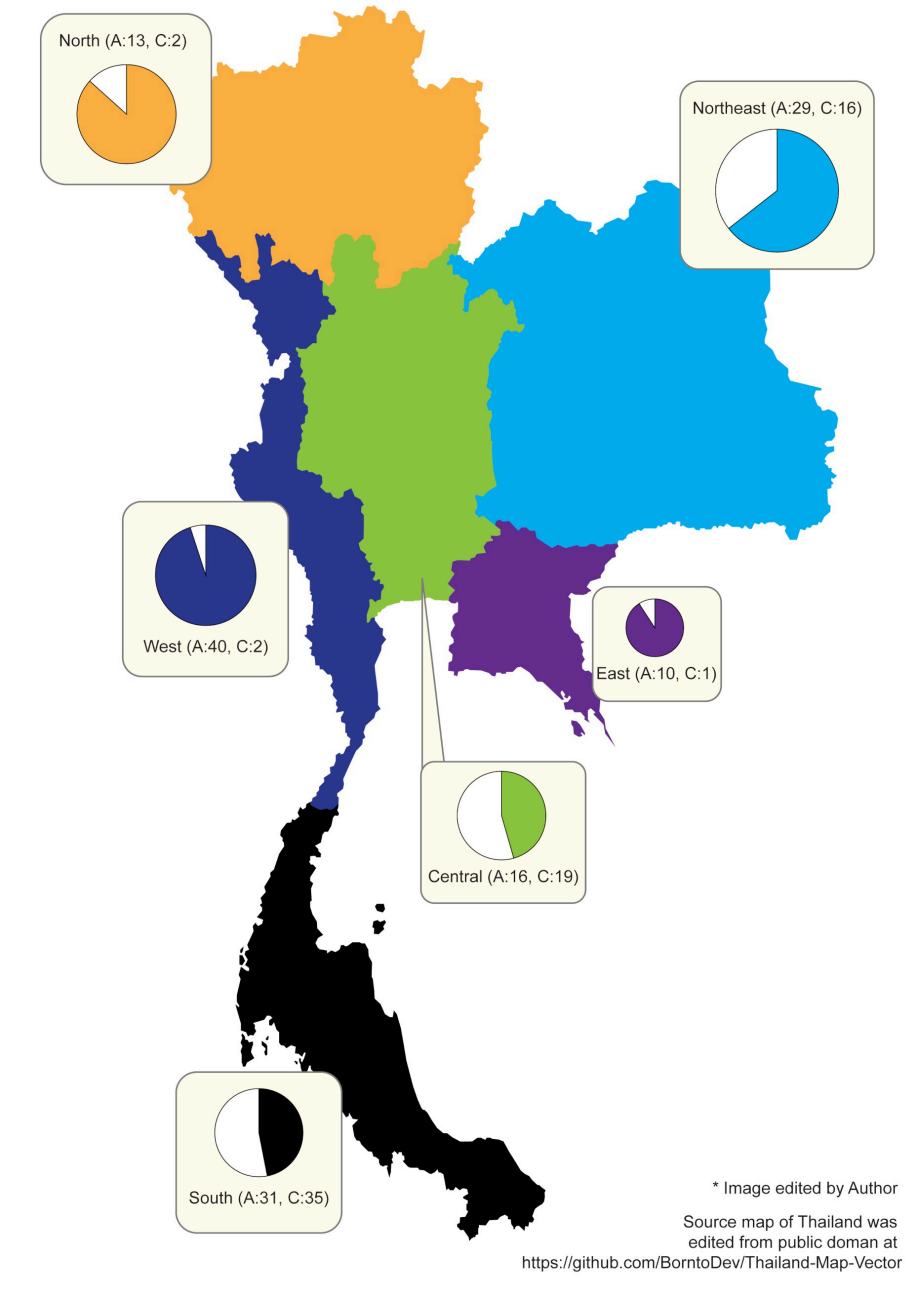
Distribution of head lice clades collected from 6 geographic regions of Thailand.

Phylogenetic analysis using data from global library together with sequences from our study revealed 6 clades as previously reported. The tree showed close connections between clade A and D, B and F, and C and E in which are also regarded as sister clades [11, 17, 18, 20–22]. Association among clades has previously been under the discussion. Co-infestations of different haplotypes as well as clades in one person were described. Therefore, interbreeding may be responsible for such connections [27, 34, 35]. For the collected head lice in our study, the phylogenetic analysis revealed two distinct clades of head lice; clade A and C (Fig 1). Clade A accounted for 65% (139/214) while clade C percentage was the remaining 35% (75/214). Region-wise, the percentage of A and C were 45.7% to 54.3% in the Central, 86.7% to 13.3% in the North, 64.4% to 35.6% in the Northeast, 47% to 53% in the South, 95.2% to 4.8% in the West, and 90.9% to 9.1% in the East (Fig 2).

**Fig 3:**
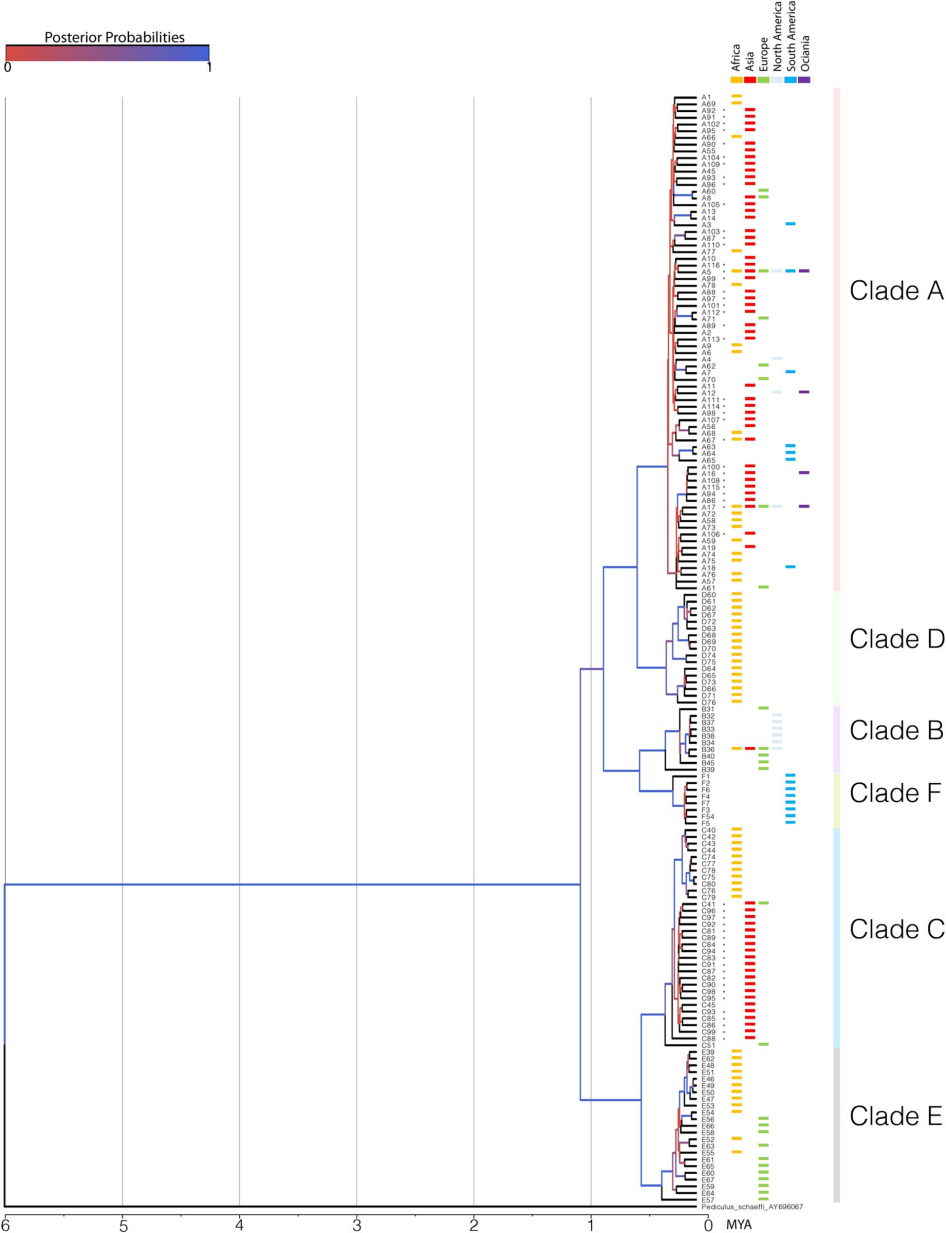
Bayesian tree. Maximum clade credibility tree was conducted based on Bayesian Method. The tree branch length is presented in MYA while the color of the branch represents the posterior probability. A total of 2097 sequences from the global library and our collected head lice were used for the analysis. The available data consisted of *cytb* gene from North America, South America, Europe, Africa, Asia, and Oceania.

From the Bayesian inference, the divergence time between *P*. *humanus* and *P*. *schaeffi*, approximately 6 MYA, was used for time tree calibration (Fig 3). The *P*. *humanus* was divided into 6 deep lineages resonating to that of the maximum likelihood tree (A, B, C, D, E, and F). The first intra-splitting of *Pediculus humanus capitis* occurred at roughly 1 MYA between cluster ABDF and cluster CE. Additionally, clusters of clade AD and BF had the most recent common ancestor around 0.9 MYA while the sister clades split later. The divergence of sister clades (D from A, F from B, and E from C) happened around the same period at 0.6 MYA. The posterior probability for the early splits was high, reaching 100% (blue), while the more recent branches hold a lower probability especially during the last roughly 0.5 MYA.

**Fig 4:**
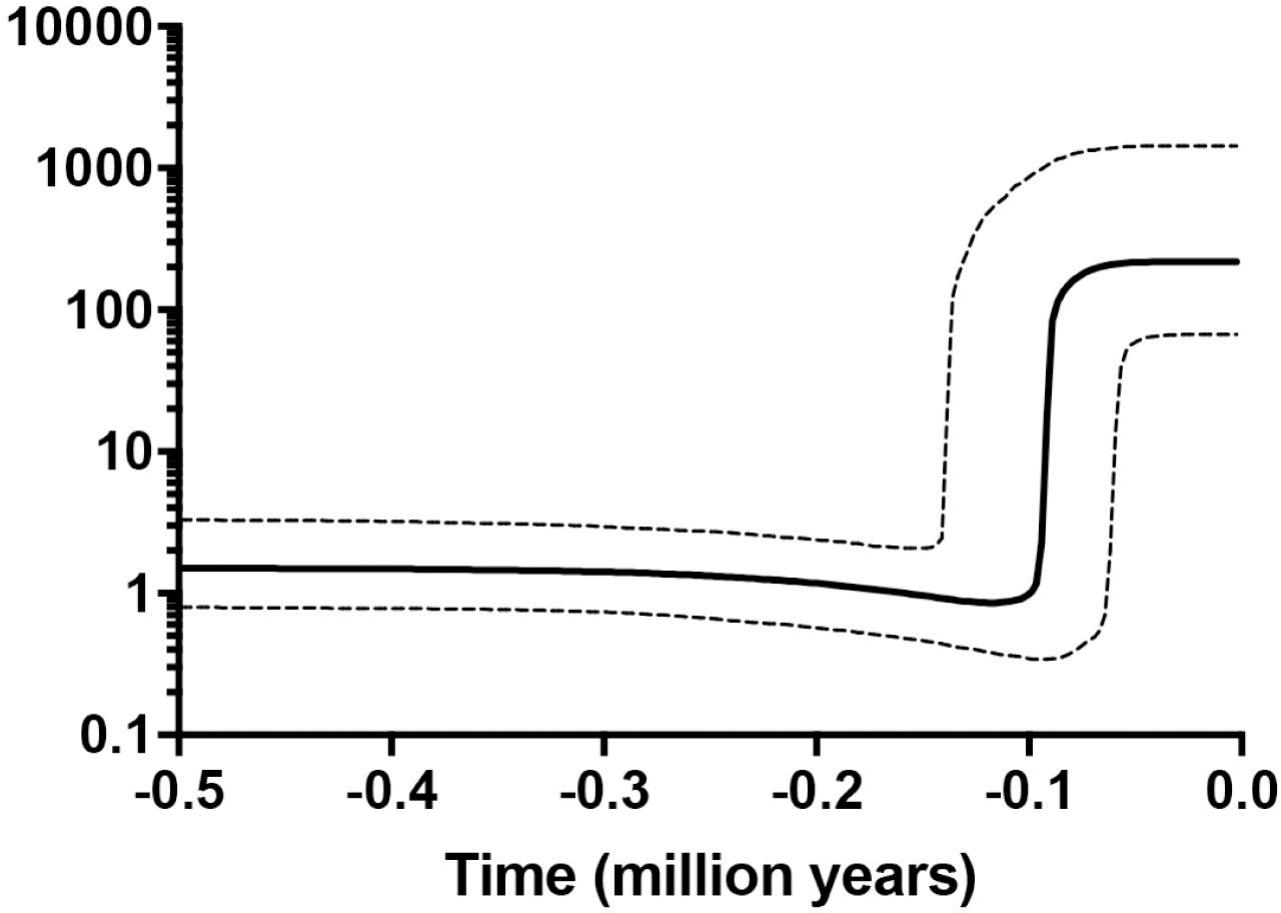
Skyline plot analysis. Bayesian skyline plot was conducted. The thick line indicated the mean of population size (*Ne*) and the thin dash line illustrated the upper and lower standard deviation (s.d.). A sharp rise in the size of the effective population was seen at approximately 100 KYA.

The skyline plot analysis exhibited a sharp increase in effective population size of head lice 100 thousand years ago (KYA) (Fig 4).

The molecular investigation from the collected head lice in our study reported 55 haplotypes, with 5 of the previously characterized and 50 novel haplotypes (Table 2). The novel haplotypes were systematically titled according to Amanzougaghene et al. in order to create a systematic lice molecular haplotypes global library. Apart from the new haplotypes, the original, A5, A16, A17, A67, and C41 were reported in our study as displayed in data in S1 Table. According to phylogenetic tree, A5 and A17 were found globally including Thailand. A16 was reported in Asia and Oceania. A67 that was found exclusively in DR Congo was surprisingly found in Thailand as well. C41 was found in Africa and Asia as well as France.

**Table 2:** Haplotype distributions of *P*. *capitis* from 6 regions of Thailand.

Regions	Mitochondria clade (*Cytb*)								
	A			C			A + C		
	Novel	Reported	Total	Novel	Reported	Total	Novel	Reported	Total
Central	4	12	16	5	14	19	9	26	35
Northern	4	9	13	0	2	2	4	11	15
Northeastern	4	25	29	2	14	16	6	39	45
Southern	8	23	31	12	23	35	20	46	66
Western	11	29	40	0	2	2	11	31	42
Eastern	1	9	10	0	1	1	1	10	11
Total	32	107	139	19	56	75	51	163	214

*Total number of novel samples was 51 with a total of namely 50 haplotypes (two were of the same haplotype)

**Fig 5:**
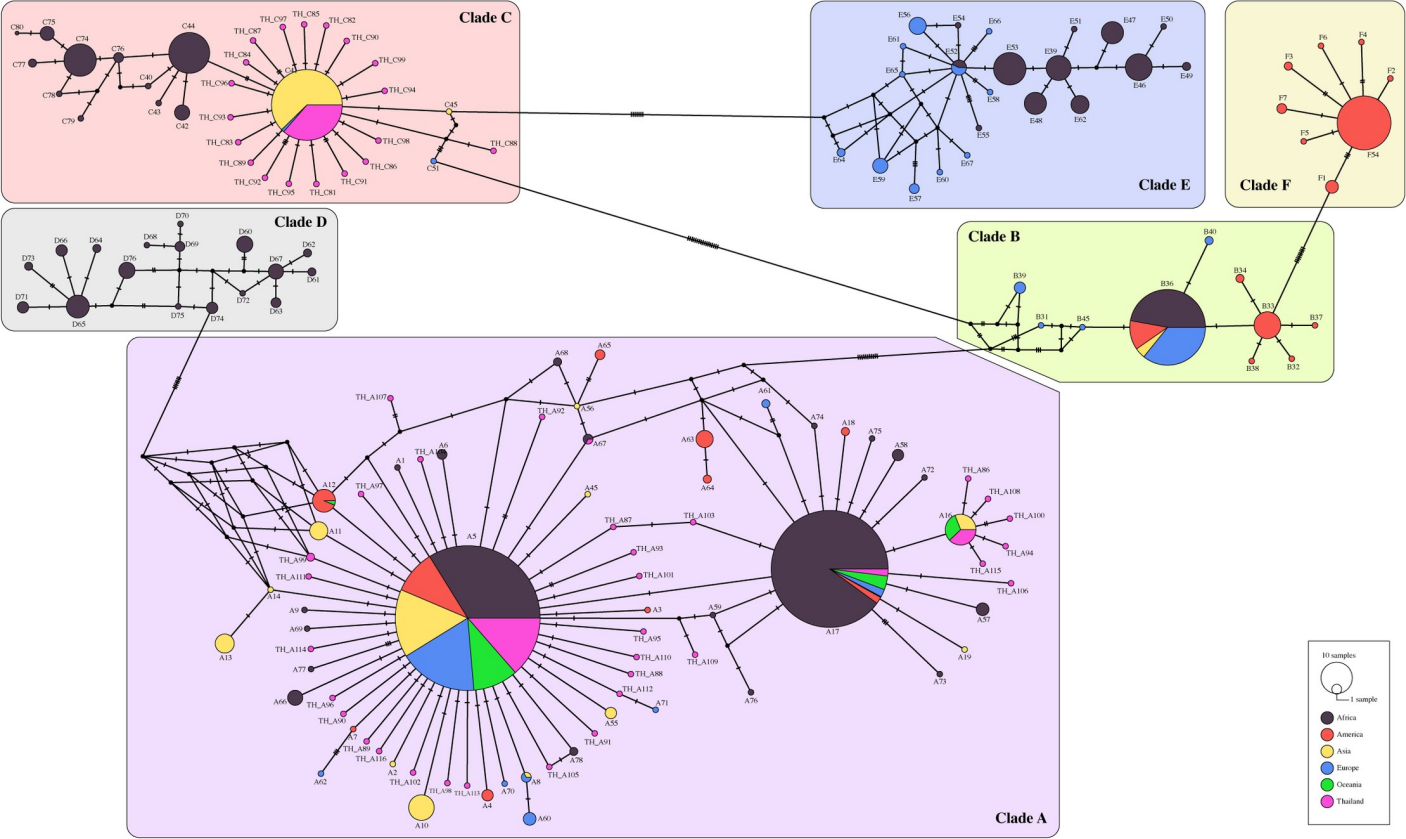
Median joining network. *cytb* sequences from our study were added in the current dataset of *P*. *capitis* for median joining network creation. The colors of the circles represent the continents. Black represents Africa, orange represents America, yellow represents Asia (excluding Thailand), blue represents Europe, green represents Oceania, and pink represents Thailand.

The newly emerged haplotypes are the result of base substitution at a variety of seventy positions. Transition mutation accounted for 74% which were base alteration from T to C (33/70) and A to G (19/70). Transversion comprised of 26% (18/70) with T mutated to C and A to G. A thorough inspection of median joining network revealed deviation by one to four mutation steps. The majority was of one-step mutation, responsible for 38 out of 50 new haplotypes. Two steps came second composing of 6/50. Three and four steps interchange were 4/50 and 2/50 respectively. It is noticed that clade F is currently restricted to only America while all clades apart from F are found in Africa. The size of the circle represents the number of registered samples. Regarding C41 haplotype, Asia excluding Thailand (yellow) has a greater number of samples compared to Thailand (pink). As an obligate human ectoparasite, the data could illustrate possible human migration between areas (Fig 5).

Of note, 24/50 novel haplotypes emerged from haplotype A5 which was the largest population in this study [40.6% (87/214)]. A16 made up of 5% (11/214) of the total specimens, A17 made up of slightly less at 3.7% (8/214), and A67 accounted for 0.47% (1/214). Five members of the novel haplotypes mutated from A16 and two from A17 with none from A67. C41 was the second most haplotypes found, accounting for 26.17% (56/214). Nineteen new haplotypes were identified from C41.

Of all collected head lice, 5 specimens from Mukdaharn (Northeastern region of Thailand) were head lice eggs because the individuals were recently infested. One specimen (A86) was identified as new and was one base different from A16. One specimen was of haplotype C41 with the remaining belonging to A5.

### Genetic diversity, haplotype analysis, and neutrality test

Haplotype diversity (Hd) measures the probability that two randomized alleles are different and nucleotide diversity (π) calculates the mean of difference of paired nucleotides of the same site [36]. We assessed a total of 214 samples of head lice collected from 6 geographic regions of Thailand using cytochrome b gene (272 bp). Of the whole population, we found 78 polymorphic sites which resulted in 55 haplotypes. Only 5 of the haplotypes were in common with the registered data leading to the identification of 50 novel haplotypes. The number of polymorphic sites were 41 (Central), 39 (Northern), 38 (Northeastern), 60 (Southern), 45 (Western), and 33 (Eastern). Hd values were similar among; Central, Southern, Western, and Eastern regions ranging from 0.629 to 0.895. For the nucleotide diversity (π), the South ranked the highest at 0.053 while the West ranked the lowest at 0.013. π results were similar among Central, Northeastern, and Southern regions of approximately 0.5. Hd and π are both relatively high in both Central and Southern regions accounting for 0.792/0.052 (Hd/ π) and 0.84/0.053 respectively (Table 3).

**Table 3:** Genetic diversity, haplotype analysis, and neutrality tests on mitochondrial *cytb* sequences.

Region	n	No. haplotype (H)	No. of polymorphic sites (S)	Average number of nucleotide differences (k)	Haplotype diversity (Hd)	Nucleotide diversity (π)	Fu and Li’s D	Tajima’s D
Central	35	13	41	15.956	0.792	0.052	-0.31321	2.16692 *
Northern	15	8	39	9.238	0.895	0.030	0.6205	-0.97973
Northeastern	45	9	38	14.467	0.629	0.047	0.39894	2.28823 *
Southern	66	30	60	16.177	0.84	0.053	-2.5093 *	0.95320
Western	42	14	45	3.973	0.692	0.013	-0.40605	-2.17998 **
Eastern	11	6	33	6.327	0.727	0.021	-2.53559 **	-2.04033 **
Total	214	55	78	13.556	0.765	0.050	-5.46811**	-0.0988

* P<0.05

** P<0.01

The metrics mentioned are used to describe the demography not the diversity. The parameters can determine the mechanism of which contributed to the observed polymorphism in a population such as the presence of neutrality evolution, also known as the coalescent theory. Neutrality of mutation refers to the shift in the genetics within a population which has no impact for the survival. The parameters used were Tajima’s D [37] and Fu & Li’s D [38]. Our data displayed significantly negative values in Eastern, Southern, and Western parts of Thailand. While the Central and Northeastern parts claimed to be significantly positive, Northern part portrayed neutrality.

### Genetic differentiation

Genetic differentiation within different populations was calculated by pairwise kst (Table 4). From the kst values, Northeastern population was not different from any regions apart from the western population.

**Table 4:** Matrix of population pairwise kst values of *P*. *capitis* from six regions of Thailand.

	Central	Northern	Northeastern	Southern	Western	Eastern
Central						
Northern	0.1199 **					
Northeastern	0.02255	0.03259				
Southern	0.00000	0.08014 *	0.02005			
Western	0.27324 ***	0.01807	0.1224 ***	0.22089 ***		
Eastern	0.13031 *	-0.01845	0.03831	0.08113 **	0.00000	

* P<0.05

** P<0.01

***P<0.001

## Discussion

Human beings are the hosts of *Pediculus humanus* while chimpanzees and bonobos are the hosts of *Pediculus shaeffi*. Since lice are obligate host-specific parasites, tracking their hosts can provide clues about their evolution, migration, and vice versa. Human lice and chimpanzee lice once had a common ancestor, roughly 6 MYA [9, 39]. The genetic divergence between present human lice and chimpanzee lice occurred concurrently with their hosts. Pediculus genera split into *Pediculus humanus* in human beings and *Pediculus schaeffi* in chimps.

Evidence of ancient human hominid inferred that the first hominid dispersal occurred 1.0 MYA, which led to the settlement of Denisovans and the spread of clade A throughout the world. Another movement occurred again 0.6 MYA, entailing the establishment of Neanderthals [11]. Interestingly, both timestamps of hominid dispersals were congruent with the splitting of major clades in our study (First wave: between cluster ABDF and cluster CE, Second wave: between sister clades; D from A, F from B, and C from E) [11]. This might imply that human spreading boosts head lice diversification. Our analysis agreed with that of Ashfaq of the earliest clade diversification timing (1.0–1.4 MYA). However, a discrepancy was observed in the most recent division (Ashfaq et al.; 0.3–0.4 MYA, Ours; 0.6 MYA). The divergence of all sister clades (D from A, F from B, and E from C) happened around the same period at 0.6 MYA not 0.3–0.4 MYA. The difference in the approach to clade division (Ashfaq et al.; A-B-C, Ours; A-B-C-D-E- F) and the addition of newer sequences from global data and our study to the analysis in our research might be the reason for the disparity [11].

Head lice of clade A existed in America long before the Columbus era (approx. 500 years ago). Several pieces of evidence pointed that body lice evolved from clade A head lice. The discovery of clade A body lice from pre-Columbus expedition period in Africa, Europe, and Asia confirmed that the dispersal of lice took place prior to the expedition [13]. The dispersion of headlice globally came much earlier according to previous analyses including ours.

Of note, lineage C and E were reported exclusively in Africa, Europe, and Asia. Their presence has not yet been identified in other lands: America and Oceania continents. As mentioned above, the most recent common ancestor of cluster ABDF and cluster CE originated in Africa. We postulated that part of the antecedents moved to other areas and later diversified as B, D, and F, while the remaining divided as clade C and E. If this assumption is valid, the related human dispersal event might have occurred earlier than 0.6 MYA.

Clade C was mainly divided into two subgroups, one derived from haplotype C41 and the other closely related to C40 and C44. The former was found in Asia, while the latter was found in Africa. Current consensus suggested that anatomically modern human began to disperse from Africa to Eurasia around 100–200 KYA. Several models indicated that the route passed through South Asia before reaching Southeast Asia [40]. The population of older original clade C head lice traveled through this route and diversified as C41. The originals that remained in Africa continued to diverge into the haplotypes of the C40 and C44 cluster. The findings explained the closer proximity of our clade C haplotypes to clade C haplotypes in South Asia.

As the evidence showed, the head lice divergence sequence was 10 times higher than that of human beings which was believed to be linked to the accelerated evolution rate [11]. We postulated that this might be related to the components of head lice short life span (high replication with substantial genetic alteration accumulation) and their potential interbreeding capacity as well as their parasitic adaptation. Globalization may also imply a window of opportunity for head lice to disperse and mingle with head lice of other clades, forming new haplotypes.

The skyline plot analysis revealed a steep rise in the population of head lice at 100 KYA. The specific time point was in parallel with 2 events; the dispersal of modern human from Africa and the exponential branching observed in our Bayesian tree [9]. It may be suggested that this wave of dispersal marked a crucial event for the head lice population in terms of both diversity and size.

Our study is the first to analyze mitochondrial *cytb* gene for the study of head lice haplotypes covering all six regions of Thailand. The regions were publicly divided according to geographical boundaries; each region differs from one another in terms of physical characteristics (climate, soil, etc.). To elucidate, the Northern region is by Laos and Burma whereas the Southern region is by Malaysia. The direction was of the same system as the world map. From the 214 collected samples in our country, we found that 65% of head lice belonged to clade A and 35% belonged to clade C. The results were congruent with the previous study of head lice in Thailand using COXI gene for molecular analysis [16]. The inclusive pattern of clade A distribution in the country also resonated with the reported data of its global distribution. Clade C head lice were reported in higher proportion in the Central, Northeastern, and Southern but contrarily lower in the Northern, Eastern, and Western areas. Clade A held the majority in the West, East, North, Northeast, and Central representing 95.23%, 90.9%, 86.66%, and 64.44% respectively. There were more clade C head lice in the Central and the South at slightly more than 50%. Moreover, our study identified fifty-five haplotypes, fifty of which were regarded as new haplotypes according to the current *P*. *humanus capitis* database namely A86-A116 and C81-C99 [17]. The robust effort to obtain head lice from various provinces to represent a region might have resulted in detection of a considerable number of novel haplotypes. We obtained head lice from a total of 26 venues (schools) throughout the country.

The majority of new haplotypes emerged from base mutation. The substitution was in the form of nucleic acid transition from C to T which is similar to prior works. Within haplogroup A, A5 was the one with highest frequency, which confirmed its globalization. Another haplotype with a considerable number of head lice in our study was A17, which is found worldwide as well [18, 20]. For A16, it has been reported in the Philippines and Australia [41]. Noticeably, the two countries, for clade A, have registered merely A5, A16, and A17; the diversity is substantially lower in comparison to our study. All of the thirty-one novel haplotypes were related to one of A5, A16, and A17. One A67 head louse, found in the East, was the same of that found in Democratic Republic of Congo (DR Congo) [42].

The majority of clade C was reported in Africa and Asia (Nepal, Pakistan, and Thailand) [11, 16, 20]. Nonetheless, a very small number head lice of clade C were also reported in France (C41) [43]. Even though clade C head lice were found in large numbers in both Africa and Asia, we found that the haplotypes from our collected lice were genetically closer to the head lice in Asia. None of the clade C haplotypes from Africa are from C41 or closer to C41 than the haplotypes obtained in our study. Countries in Asia (Nepal, Pakistan, and Thailand) collectively accounted for the vast majority of haplotype C41 worldwide. In our study, we reported 56 head lice of the existing haplotypes C41. The other remaining clade C novel haplotypes (C81-87 and C89-C99) were closely linked to C41. The novel haplotype C88 was at closest proximity to C45.

In Thailand, clade A is ubiquitously distributed whereas clade C is clustered in Central, Northeastern, and Southern regions. Haplogroup C was absent in the Eastern, Northern, and Western regions. As for the world, clade C is restricted to only Africa and Asia (Nepal, Pakistan, and Thailand), with a small number found in Europe. Prehistorical evidence suggested that modern human travelled through South Asia to Southeast Asia. More recent history of migration was traced in a bid to find the association of clade C head lice population in other countries and Thailand. Referring to a historical material, we found that Nepalese who fought with the British in World War II remained in Burma after the war ended in 1945 AD. They confidentially settled at the border between Thailand and Burma where they mined for ores. After ores ran out, most of the Nepalese traveled to tourist cities for jobs; Bangkok and cities along the beaches in the South. As there is still no clear evidence, we suggested that the hypothesis was just another possible presupposition explaining the spread of head lice clade C to the Central and South of Thailand. The conflict is the absence of clade C population in the west, but we assumed that this might be due to either the issue of sampling area coverage or the settlers’ self-area restriction [44, 45]. Data of Pakistani population in Thailand is non-specific. Therefore, further study of head lice clade in Burma might help confirm or reject the aforementioned presupposition. The current database (not displayed) exhibited that Pakistan and Nepal were the only two countries apart from Thailand to be populated with C41 haplotypes in Asia, accounting for 79 and 17 samples, respectively. Therefore, it is assumed that Thailand and South Asia head lice population might be related in the past. We did not expect any correlation of African and Thai population as the clade C haplotypes of two regions were distinct from each other as previously mentioned. According to Anglo-Siamese 1909 Treaty, Siam (Thailand) had to transfer the present-day Northern Malaysia land to Britain in exchange for other British support [46]. This implied that the population in the far South of Thailand and North of Malaysia were once one country without borders allowing interconnection. However, head lice of clade A, B, and D not C were discovered in Malaysia [22]. The absence of clade C in Malaysia and clade B and D in Thailand might be a consequence of inadequate sampling or head lice migration of a later time.

Higher haplotype diversity and lower nucleotide diversity in our study suggested that there was a recent divergence in the studied population. The results were congruent with our median joining network showing a majority of novel haplotypes differed from the main haplotype for only one to two bases. Neutrality indices were on the negative side for the Eastern, Southern, and Western parts of Thailand suggesting selective sweep or rapid population expansion. Tajima’s D is beneficial to isolate the mutation frequency within a constant number population. Central and Northeastern regions showed positive figures, which means the population experienced high polymorphism, which could be due to any factors jeopardizing the population, such as the usage of pediculicide. High migration rate can also be accounted for this incident. According to Thai Census conducted between 2010–2014 [47], the data showed that Northeastern region had the greatest number of migrants from other geographical regions, while Bangkok in the Central is the country’s metropolis renowned for its urban centralization [37]. Northern results were not statistically significant (the estimates are not significantly different from 0) and could be interpreted as population evolving as per mutation-drift balance or no evidence of selection [37].

Genetic differentiation depicts gene flow between populations. In the present study, the pairwise kst exhibited no differentiation between Northeastern and other regions. As previously mentioned, Northeastern population received the highest migrants between 2010 and 2014. It is assumed that the mix among people of different populations had in part led to non-differentiation results. The main purpose for moving was work.

Our limitation is the relatively low number of samples in some regions such as the East and the North. Moreover, studies about head lice genetic polymorphism is not widespread in Asia and the specimens retrieved in each country were in extreme diversity. The other issue is the use of COXI gene instead of *cytb* gene in former studies. This leads to the discrepancies in the evaluation of the data to indicate authentic diversity along with the difficulties to come up with a reliable statistical association. More studies of head lice in neighboring countries are required to predict the trend in the area to provide a better understanding of head lice evolution in Asia. As of now, our study detected numerous both common and divergent novel haplotypes. Furthermore, we also identified head lice haplotypes that were the same of those reported in South Asia. According to the associated history of human migration, it might be inferred that Thailand has had a long history of head lice.

Our work contributes to the study of phylogeography distribution of head lice for a deeper understanding of human evolution and migration. The extent to which Thai head lice population relate or differ from that of other continents in terms of molecular data is the question to be further explored. Broader geographic molecular analysis of head lice together with population statistical analysis could help generate the mapping of population migration between Thailand and other global populations. Thus far, there has been no studies that examine the correlation between head lice clades/haplotypes, the permethrin resistance gene, and the presence of bacteria in head lice. Establishing a link among these three crucial facets of head lice might be of great value for future head lice controls. We hope our study could serve as the essential basis for future head lice researches [42].

## Supporting information

S1 Table Haplotypes of *Pediculus humanus capitis* in Thailand. (PDF)
