# Supplementary material for: Molecular analysis of mitochrondrial cytb of Pediculus humanus capitis in Thailand revealed potential historical connection with South Asia
Source: PLoS One. 2021 Sep 7;16(9):e0257024. doi: 10.1371/journal.pone.0257024 (PMC8423300; doi:10.1371/journal.pone.0257024)
Supplement: S1 Table — (PDF) [file pone.0257024.s001.pdf]

S1 Table Haplotypes of *Pediculus humanus capitis* in Thailand

Clade A (Reported)

Haplotype	Central	North	Northeast	East	South	West
**A5**	10	2	24	6	21	24
**A16**	0	3	1	1	1	5
**A17**	2	4	0	1	1	0
**A67**	0	0	0	1	0	0

Clade A (Novel)

Haplotype	Central	North	Northeast	East	South	West
**A86**	0	0	1	0	0	0
**A87**	0	0	0	0	0	1
**A88**	0	0	0	0	0	1
**A89**	0	0	0	0	0	1
**A90**	0	0	0	0	0	1
**A91**	0	0	0	0	0	1
**A92**	0	0	0	0	0	1
**A93**	0	0	0	0	0	1
**A94**	0	1	0	0	0	0
**A95**	0	0	1	0	0	0
**A96**	0	0	1	0	0	0
**A97**	0	0	1	0	0	0
**A98**	0	0	0	0	0	1
**A99**	0	0	0	0	0	2

Clade A (Novel) (Continue)

Haplotype	Central	North	Northeast	East	South	West
**A100**	0	0	0	0	0	1
**A101**	1	0	0	0	0	0
**A102**	1	0	0	0	0	0
**A103**	1	0	0	0	0	0
**A104**	1	0	0	0	0	0
**A105**	0	0	0	0	1	0
**A106**	0	0	0	1	0	0
**A107**	0	1	0	0	0	0
**A108**	0	1	0	0	0	0
**A109**	0	1	0	0	0	0
**A110**	0	0	0	0	1	0
**A111**	0	0	0	0	1	0
**A112**	0	0	0	0	1	0
**A113**	0	0	0	0	1	0
**A114**	0	0	0	0	1	0
**A115**	0	0	0	0	1	0
**A116**	0	0	0	0	1	0

Clade C (Reported)

Haplotype	Central	North	Northeast	East	South	West
**C41**	14	2	14	1	23	2

Clade C (Novel)

Haplotype	Central	North	Northeast	East	South	West
**C81**	1	0	0	0	0	0
**C82**	1	0	0	0	0	0
**C83**	1	0	0	0	0	0
**C84**	1	0	0	0	0	0
**C85**	0	0	0	0	1	0
**C86**	0	0	0	0	1	0
**C87**	0	0	1	0	0	0
**C88**	0	0	1	0	0	0
**C89**	0	0	0	0	1	0
**C90**	0	0	0	0	1	0
**C91**	0	0	0	0	1	0
**C92**	0	0	0	0	1	0
**C93**	0	0	0	0	1	0
**C94**	0	0	0	0	1	0
**C95**	0	0	0	0	1	0
**C96**	0	0	0	0	1	0
**C97**	0	0	0	0	1	0
**C98**	1	0	0	0	0	0
**C99**	0	0	0	0	1	0
